# Supplementary material for: Polymeric Nanoparticles Based on Tyrosine-Modified, Low Molecular Weight Polyethylenimines for siRNA Delivery
Source: Pharmaceutics. 2019 Nov 12;11(11):600. doi: 10.3390/pharmaceutics11110600 (PMC6920781; doi:10.3390/pharmaceutics11110600)
Supplement: Supplementary file 1 [file pharmaceutics-11-00600-s001.pdf]

pharmaceutics

Supplementary Materials: Polymeric Nanoparticles Based on Tyrosine-Modified, Low Molecular Weight Polyethylenimines for siRNA Delivery

Alexander Ewe, Sandra Noske, Michael Karimov and Achim Aigner

Table S1. siRNA sequences.

siRNA	Sequence (5′ – 3′)		
siLuc2 [(a)]	*sense*	CGUACGCGGAAUACUUCGA dTdT	Dharmacon Horizon Discovery, Lafayette, CO, USA
	antisense	UCGAAGUAUUCCGCGUACG dTdT	
siLuc3 [(b)]	*sense*	CUUACGCUGAGUACUUCGA dTdT	Eurogentec, Seraing, Belgium
	antisense	UCGAAGUACUCAGCGUAAG dTdT	
siEGFP	*sense*	GCAGCACGACUUCUUCAAG dTdT	Eurofins Genomics, Ebersberg, Germany
	antisense	CUUGAAGAAGUCGUGCUGC dTdT	
siPLK1	*sense*	GAAGAUGCUUCAGACAGAU dTdT	Bianoscience, Gera, Germany
	antisense	AUCUGUCUGAAGCAUCUUC dTdT	
siSurvivin	*sense*	GAAUUAACCCUUGGUGAAU dTdT	Eurogentec, Seraing, Belgium
	antisense	AUUCACCAAGGGUUAAUUC dTdT	
siBcl-2	*sense*	AACAUCGCCCUGUGGAUGACU dTdT	Dharmacon Horizon Discovery, Lafayette, CO, USA
	antisense	AGUCAUCCACAGGGCGAUGUU dTdT	
siGAPDH #1	*Sense*	AUUCCAUGGCACCGUCAAG dTdT	Eurogentec, Seraing, Belgium
	antisense	CUUGACGGUGCCAUGGAAU dTdT	
siGAPDH #2	*sense*	CCUCAACUACAUGGUUUAC dTdT	Eurogentec, Seraing, Belgium
	antisense	GUAAACCAUGUAGUUGAGG dTdT	
siGAPDH #3	*sense*	UCCAUGACAACUUUGGUAU dTdT	Eurogentec, Seraing, Belgium
	antisense	AUACCAAAGUUGUCAUGGA dTdT	
siUbb	*sense*	GGCCAAGAUCCAAGAUAAA dTdT	Eurogentec, Seraing, Belgium
	antisense	UUUAUCUUGGAUCUUGGCC dTdT	
siLuc3-Atto488	*sense*	Atto488-CUUACGCUGAGUACUUCGA dTdT	Eurogentec, Seraing, Belgium
	antisense	UCGAAGUACUCAGCGUAAG dTdT	
siAlexa647		Scrambled, Undisclosed by the vendor	Qiagen, Hilden, Germany

[(a)] siLuc2 served as negative control siRNA for luciferase knockdown experiments.

[(b)] siLuc3 is the specific luciferase siRNA, but served as negative control siRNA in all other knockdown experiments.

Table S2. RT-qPCR primer sequences.

Primer		Sequence (5′ – 3′)	
GAPDH	for	GGT GTG AAC CAT GAG AAG TAT GA	Bianoscience, Gera, Germany
	rev	GAG TCC TTC CAC GAT ACC AAA G	
RPLP0	for	TCT ACA ACC CTG AAG TGC TTG AT	Eurofins Genomics, Ebersberg, Germany
	rev	CAA TCT GCA GAC AGA CAC TGG	
Survivin	for	TGA TGA GAG AAT GGA GAC AGA G	Bianoscience, Gera, Germany
	rev	ACA GCA GTG GCA AAA GGA G	
PLK1	for	ATC TTC TGG GTC AGC AAG TG	Eurofins Genomics, Ebersberg, Germany
	rev	GCC GTC ACG CTC TAT GTA CT	

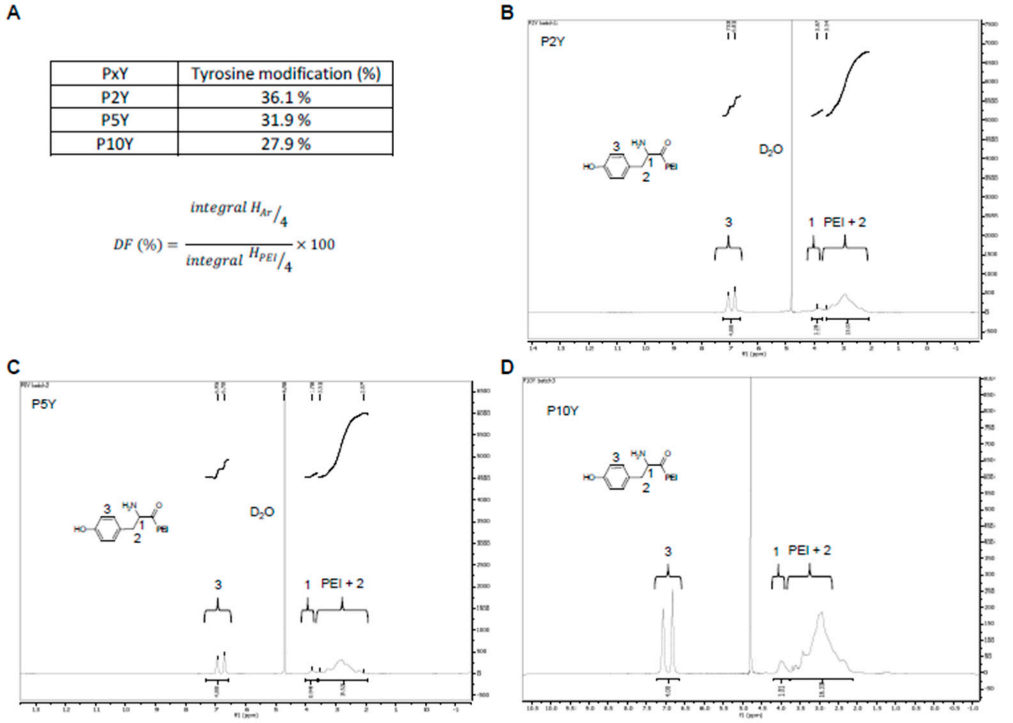

Figure S1. Degrees of tyrosine grafting and original ^{1}H-NMR spectra.

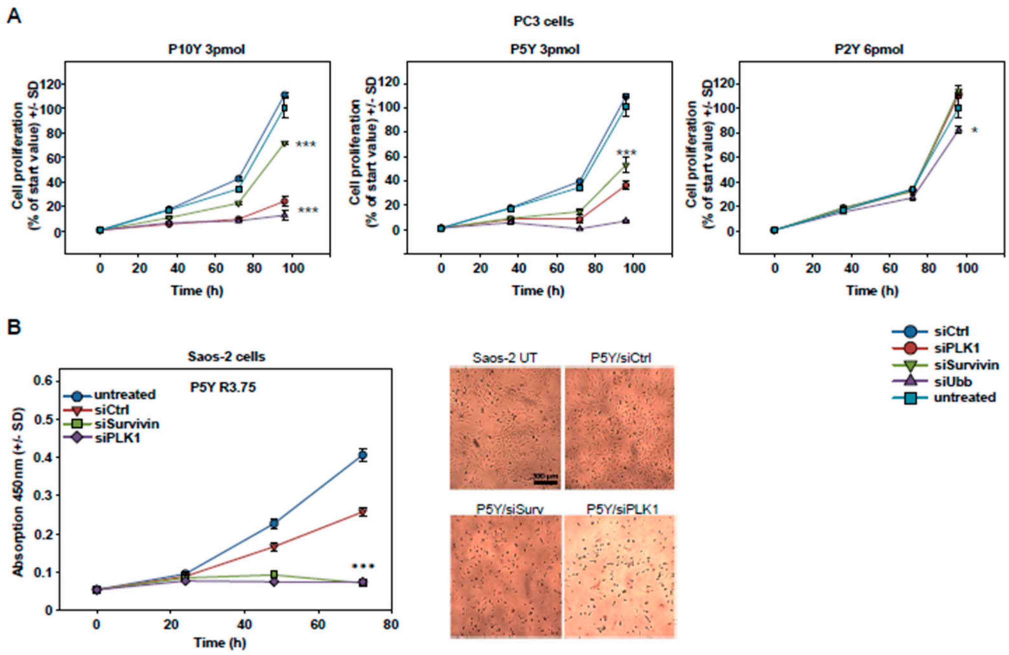

Figure S2. Additional data to Figure S1.

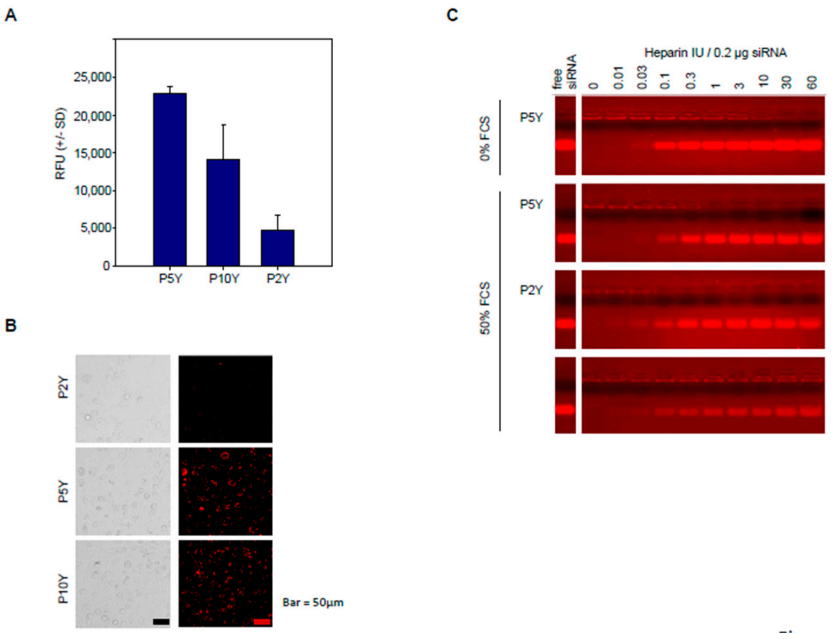

Figure S3. (**A**,**B**): Cellular siRNA uptake, (**C**) complex stabilities.

© 2019 by the authors. Submitted for possible open access publication under the terms and conditions of the Creative Commons Attribution (CC BY) license (http://creativecommons.org/licenses/by/4.0/).
